# Comparing the effectiveness of a group-directed video instruction versus instructor-led traditional classroom instruction for learning cardiopulmonary resuscitation skills among first-year medical students: A prospective randomized controlled study

**DOI:** 10.3205/zma001566

**Published:** 2022-09-15

**Authors:** Mexmollen Marcus, Ariff Arithra Abdullah, Junainah Nor, Tuan Hairulnizam Tuan Kamauzaman, Nicholas Tze Ping Pang

**Affiliations:** 1 Universiti Sains Malaysia, School of Medical Sciences, Kelantan, Malaysia; 2 Universiti Malaysia Sabah, Faculty of Medicine and Health Sciences, Sabah, Malaysia; 3 Hospital Universiti Sains Malaysia, Kelantan, Malaysia

**Keywords:** cardiopulmonary resuscitation, basic life support, teaching materials, clinical skills, medical education

## Abstract

**Introduction: **Bystander cardiopulmonary resuscitation (CPR) training is inconsistent among students and the public. Existing CPR teaching courses are costly, time-consuming, and inconsistent. This study aimed to determine the association between overall CPR competency and two teaching modules, a group-directed video instruction module versus an instructor-led traditional classroom instruction module.

**Methods: **This randomized prospective interventional study involved first year medical students of Universiti Sains Malaysia Health Campus from November 2018 until January 2019. Pass-fail scores representing the overall CPR, individual skill performance, and willingness to perform CPR for strangers and family members were collected. Factors associated with reluctance to perform CPR were assessed in a questionnaire.

**Results:** A total of 99 participants were included, 50 in the group-directed video instruction as the intervention module and 49 in the traditional classroom instruction as the control module. There was no statistical significance between the pass and fail outcomes for both video module (*p*=0.436). Participants in both modules performed similarly in 8 out of 12 individual CPR skills. There was a significant difference in the distribution of skill scores between the pass and fail outcomes (*p*=<0.001). The intervention module is non-inferior compared to the control module, in relation to CPR willingness rates for strangers (*p*=0.999) and family members (p=0.117) after the training.

**Conclusions: **The group-directed video self-instruction method is as effective as the instructor-led traditional classroom method to help participants to be competent and willing to perform CPR. It can be used as an independent or supplementary teaching tool for first-time learners and refreshers, especially in a group setting when teaching materials are limited.

## Zusammenfassung

**Einleitung: **Die Ausbildung zur Herz-Lungen-Wiederbelebung (HLW) durch Laien ist bei Schülern und in der Öffentlichkeit uneinheitlich. Bestehende HLW-Kurse sind kostspielig, zeitaufwendig und uneinheitlich. Ziel dieser Studie war es, den Zusammenhang zwischen der allgemeinen HLW-Kompetenz und zwei Unterrichtsmodulen zu ermitteln: einem gruppengeleiteten Video-Unterrichtsmodul und einem von einem Ausbilder geleiteten traditionellen Unterrichtsmodul.

**Methoden: **An dieser randomisierten, prospektiven Interventionsstudie nahmen Medizinstudenten des ersten Studienjahres der Universiti Sains Malaysia Health Campus von November 2018 bis Januar 2019 teil. Pass-Fail-Scores, die die allgemeine HLW, die individuellen Fähigkeiten und die Bereitschaft zur Durchführung einer HLW bei Fremden und Familienmitgliedern darstellen, wurden erfasst. Faktoren, die mit der Zurückhaltung bei der Durchführung der HLW zusammenhängen, wurden in einem Fragebogen bewertet.

**Ergebnisse: **Insgesamt wurden 99 Teilnehmer eingeschlossen, 50 in der gruppengeleiteten Videoschulung als Interventionsmodul und 49 in der traditionellen Präsenzschulung als Kontrollmodul. Es gab keine statistische Signifikanz zwischen den bestandenen und nicht bestandenen Ergebnissen für beide Videomodule (*p*=0,436). Die Teilnehmer beider Module zeigten bei 8 von 12 einzelnen HLW-Fertigkeiten ähnliche Leistungen. Es bestand ein signifikanter Unterschied in der Verteilung der Fertigkeitsergebnisse zwischen den bestandenen und den nicht bestandenen Ergebnissen (*p*=<0,001). Das Interventionsmodul ist dem Kontrollmodul nicht unterlegen, was die Bereitschaft zur Wiederbelebung von Fremden (p=0,999) und Familienmitgliedern (*p*=0,117) nach dem Training betrifft.

**Schlussfolgerungen: **Die gruppengeleitete Video-Selbstinstruktionsmethode ist ebenso wirksam wie die von einem Ausbilder geleitete traditionelle Unterrichtsmethode, wenn es darum geht, den Teilnehmern zu helfen, kompetent und bereit zu sein, eine HLW durchzuführen. Sie kann als unabhängiges oder ergänzendes Lehrmittel für Erst- und Auffrischungsschüler eingesetzt werden, insbesondere in einem Gruppenumfeld, in dem die Unterrichtsmaterialien begrenzt sind.

## Introduction

Heart disease is still the leading cause of death for the non-communicable diseases’ category in the modern world and sudden cardiac arrest is the third leading cause of death in industrialized nations [1], [2], [3]. This makes out-of-hospital cardiac arrests (OHCA) a growing concern [3], [4], [5], [6]. Numerous studies have shown that CPR done at the scene of an OHCA increases a patient’s survival however rates of bystander CPR varies significantly across the globe [4], [7], [8], [9].

Collective data shows that amount of people trained in cardiopulmonary resuscitation (CPR) among the public varies from 30% to 60%, and even lesser numbers have actually performed CPR in real-life [10], [11], [12], [13], [14]. Training cost, time allocation, logistics, lack of learning motivation, and heavy information load all contribute towards the difficulties of learning CPR [15], [16]. Reluctance in performing CPR among those who already had training also needs to be addressed [10], [11], [17]. These points out towards the need to emphasize on not only the initiation of the CPR training, but also its continuous refreshers. 

In Malaysia, formal CPR training is based on the AHA Basic Life Support (BLS) training. Students from the medical faculty receive formal BLS training only once in their syllabus, and this is not adequate to equip them with the necessary CPR skills upon graduation [18], [19], [20], [21]. Junior doctors, or house officers are expected to handle, or at least initiate resuscitations in the hospital on their first day, but most are inept at performing CPR due to lack of exposure during their undergraduate years [22], [23], [24], [25]. They are also likely to be reluctant to provide bystander CPR outside of the hospital setting due to social stigma, and fear of doing mistakes [18], [26], [27]. Long working hours and expensive CPR training fees hindered them from attending BLS courses. Without proper training, junior doctors performing CPR in distant hospitals where there is no supervision from a senior colleague have very little opportunity to correct their techniques [28], [29], [30], [31], [32]. CPR knowledge and performance are also poor among doctors in certain fields where patients are not expected to deteriorate and require resuscitation [33]. Given these scenarios, BLS training has become a mandatory component in medical undergraduate curriculums, to train and educate them regarding correct technique and proper attitude towards performing CPR.

BLS courses in Malaysia are usually catered for participants in which they will be divided into groups, and each group will work with a mannikin. The size of the groups are determined by the amount of mannikins provided by the course organizer. This poses a problem in outreach BLS programmes, especially in hard to access rural places where transportation of mannikins is limited. Most medical universities and hospitals in Malaysia also have limited amount of training mannikins. Most of the research studying correlation between CPR skill acquisition and training module were able to provide the participant with a training mannikin each [34], [35], [36], [37]. However, this ideal scenario is not achievable to all teaching institutions especially in underdeveloped and developing nations. This shortcoming was also addressed in the previous studies where success needs to be replicated in group settings [38], [39].

Alternative approaches to the traditional CPR learning and the pre-existing video modules are needed to help alleviate the problem. Previous studies have successfully demonstrated when used by a single learner the instructional video was able to result in a competent overall CPR performance , despite slight variations in individual skill performance when the outcome was compared to the control group [34], [36], [40], [41], [42], [43]. However as previously mentioned, underprivileged populations may not benefit from that approach due to lack of resources. Our main goal in this study is to prove that video learning for CPR skills acquisition is also applicable when used in a group-directed setting. We believe also that with the interaction within a group, peer-to-peer guidance and feedback may improve the individual skill performance of members as highlighted in outcomes of the previous studies.

## Methods

This randomized prospective interventional study from November 2018 until January 2019 involved first year students of the Universiti Sains Malaysia (USM) health campus. 

Prior to participant recruitment, two modules for teaching CPR were created.

### 1. Modules

The control module is the traditional classroom instruction module (see attachment 1 , point H). It is a 4-hour CPR simplified classroom lecture and practical session based on the standard AHA 2015 BLS course. We presented only the complete steps of how to perform BLS [44]. The delivery style 2-hour lecture was dependant on the trainer, including the time allocated for questions from the participants. Upon completion of the lecture, participants will be guided in the practical CPR sessions for the remaining 2 hours. 

The intervention method is the video instruction module based on the standard AHA 2015 BLS, which emphasises on the full demonstration of the complete steps of BLS – emphasizing on chest compression and ventilation techniques delivered via a series of pre-recorded videos [44]. The videos were supplemented with captions, subtitles, and audio cues to ensure the participant received both audio and visual information. The videos were recorded in our simulation room using similar mannikins used in the study. The video module was approximately 30 minutes in duration, in which time was also allocated for the participants to perform skills together along with the recording. Groups may choose to repeat the video up to a maximum of 4 times up to 2 hours, and they may pause and rewind the video to focus on a certain part. 

After completion of both modules, all participants were ushered into the holding room where they will be randomized to different examiners for assessment. Standard Laerdal^®^ Resusci-Anne Simulator manikins were used in practical sessions of both modules. 

#### 2. Scoring sheets

Two scoring sheets for assessment were used. The first sheet is a 5-point Likert score sheet to subjectively assess the overall CPR performance of the participants (see attachment 1 , point A). The second scoring sheet will determine adequacy or inadequacy in 12 elements in CPR (see attachment 1 , points B, C). Both scoring forms have already been validated by a pilot study [38], [40], [45] and been used as a basis of various similar studies. After revision of the forms with our panel of content experts, we noticed that 2 items from the scoring sheet which were “initial opening of airway” and “initial breaths before compression” are already obsolete in regards to latest CPR guidelines, thus we opted to use the validated 2015 AHA BLS Checklist which is used to globally to assess overall CPR competency [44].

A pre-validated questionnaire sheet [40] was used to gather demographic data and the participants’ opinions regarding the course duration, course content, and the time spent doing the practical sessions in the modules. The questionnaire also required the participants to gauge their willingness to perform CPR for strangers and family members, before and after the training. These forms were given to the participants before the intervention to get the pre-test data, and completed after the reassessment for the post-test data.

#### 3. Instructor selection criteria

We recruited a total of 10 post-graduate doctors, all certified and accredited BLS providers, from the Emergency Medicine department to be the instructors for the study. Each instructor only fulfilled one role to prevent bias – 1 trainer and 2 facilitators for the classroom module, 1 invigilator for the video module, and 6 examiners. Instructors were also briefed regarding their roles and accustomed them to the scoring sheets in a separate meeting prior to the study.

#### 4. Training plan

On the day of the study, participants who were randomized to the classroom module were all gathered in the main simulation room for training. Participants of the video module were again randomly assigned into groups of eight by block randomization method [46]. Groups were assigned to separate tutorial rooms in the Emergency Department, each equipped with a projector, a computer and a practice manikin. Each group viewed the video module together and followed the instructions shown in the video. 

After completion of both modules, all participants were ushered into the holding room where they will be randomized to different examiners for assessment. Standard Laerdal^®^ Resusci-Anne Simulator manikins were used for practice in both modules.

#### 5. Bias reducation 

Few measures were taken to reduce bias after the training. First, the study setting included several separate rooms, 6 were used for the video module. The main simulation room was used for the classroom module. Second, a holding room was prepared to hold the participants before the post-training assessment. Appropriate media was played in the holding room to prevent the incidental transfer of sound from the other rooms. Direct supervision under the instructors in the holding room was to prevent participants from having discussions among them. Both subjects’ and instructors’ informed consent included a non-disclosure agreement. Study materials and rooms were concealed from view when not in use.

#### 6. Post-training assessment

Following the previous studies, we only assessed the post-training CPR outcome since the participants have no prior training or knowledge about BLS, we believe that they will be unable to pass the pre-training assessment.

For the post-training assessment, participants from both groups were randomized to examiners using an online randomizer [46]. A pair of examiners were assigned to each assessment room. Examiners were blinded to the module that was assigned to each participant. At the start of each assessment, each participant was briefed on the scenario (see attachment 1 , point G). Similar Laerdal^®^ Resusci-Anne Simulator manikins were used as an aid to evaluating the candidates' CPR performance. 

During the assessment, examiners scored from 1-5 for the overall subjective CPR performance of the participant. Both examiners in the assessment room must agree on the general score that was given to the participant. Examiners also assessed the individual skill adequacy with a “pass” or “fail” remark. After the assessment, examiners held private feedback sessions to debrief the participants about their performance.

Lastly, the participants completed a questionnaire (see attachment 1 , point F) in which they were asked about their demographic data, and prior CPR training, if any as well as their opinion regarding the study. We included the participants who failed in the initial assessment into the questionnaire as our second objective aims to determine if willingness to perform CPR is dependent on different teaching methods, and not correlating it with participants’ competency.

#### Statistical analysis

Data were explored and analysed using Statistical Package for the Social Sciences (SPSS) version 24.0 (SPSS, Inc, Chicago, IL, USA). Categorical variables were presented as frequency and percentage. Continuous variables were presented using mean and standard deviation. The Chi-square and Fisher exact tests were used to find an association between modules and pass-fail outcomes. Mann-Whitney U Test was used to find difference between sum of individual skill score and the pass-fail outcomes. The McNemar test was used to find an association between willingness improvement rates before and after the training. Statistical significance was taken as a p-value of <0.05.

The required sample size was derived from two early studies by Todd et al. [38], [46]. They described a power calculation with a 2-sided α-level of 0.05 and β-level of 0.2 and found that an estimate of 27 subjects per group to be statistically significant. 

## Results

Out of the 210 first-year students we approached, 118 of them consented and signed up for the study. However, only 99 participants were included in the study as 19 (16%) of them were excluded from the analysis due to their past exposure to CPR. 

For the overall performance CPR of the two modules, we concluded that there was no significant statistical association between the type of training module and the pass-fail outcome (*p-value=0.436*). 

Classroom module participants scored better than video module participants in four individual CPR skills. There is a statistically significant association between the classroom module and better performance in skills 2,5,9,10. However, for the other eight individual CPR skills, we concluded that there was no significant statistical association between the type of training module and the pass-fail outcomes (*p-value>0.05*). 

Sum total of individual skills for those who passed (Mdn=11) were higher than those who failed (Mdn=6). A Mann-Whitney U Test indicated that this difference was statistically significant U(N_pass_=92, N_fail_=7,)=641.00, z=4.505, *p=<.001*, thus corroborating our initial pass-fail assessment.

We also determined if the modules had a positive impact on the participants’ willingness to perform CPR in both scenarios. Both methods are statistically proven to demonstrate an increase in CPR willingness rates after training for both scenarios (*p-value<0.001*). 

Moreover, the video module is non-inferior to the classroom module, in relation to CPR willingness rates after training for both situations “CPR for strangers” (*p-value=0.990*) and “CPR for family” (*p-value=0.117*). Both these results support our null hypotheses.

Participants who feel they were unable to perform CPR stated that the lack of proper training was the main hindrance factor. Lack of proper training was the reason cited for CPR reluctance in real-life situations, despite the module being thought to be efficient. An exception in the classroom module, where 60% of them fear of contracting an infection through CPR from strangers even after the training. In our study, 92% of the video module participants would choose to learn CPR via this method again, and they would recommend it to others. Eighty-two per cent of them thought that the module was sufficient (see table 1 (Tab. 1), table 2 (Tab. 2), table 3 (Tab. 3), table 4 (Tab. 4) and table 5 (Tab. 5)).

## Discussion

Previous studies comparing self-directed video-based learning to traditional class learning for acquiring CPR skills found that video learning was superior to the classroom method. In our study, we observed group-directed video-based learning for previously untrained individuals and compared the end results with a traditional classroom instruction module.

Our group-directed video-based learning yielded similar effectiveness to the traditional classroom instruction in producing optimal overall CPR competency. However, the latter method produced better results in certain specific skills, namely locating the landmark for compression point, and compression speed. 

For our study, we decided to emphasize the time spent to demonstrate these skills in our video recording, knowing that this was the limitation in previous self-directed studies [34], [40], [45]. Despite the modifications done, our results still reflected the results obtained from the self-directed video learning studies. The skills performed poorly in the video group (to locate the precise compression point and perform optimal compression metrics) ensure high-quality CPR, thus making it the primary component in influencing a victim's survival from cardiac arrest [44], [47]. Based on this finding, we concluded that the traditional classroom instruction with trainer feedback maybe be better suited for demonstrating skills that require a student to locate certain landmarks on the human body, and to perform compression to the correct tempo. However, it is imperative to mention that the other skills are of equal importance to ensure the victim's highest possible chance of survival, as evidenced by their inclusion in standardized BLS assessment modules [44], [47], [48]. 

For skill *“shake and shout”*, the participants were required to firmly tap the shoulders of the victim and shout loudly to get their attention. This skill was rather straightforward and was demonstrated clearly in the video and was repeated multiple times. However, the video group underperformed this skill during the assessment. We theorized that this could be an isolated finding, as in most of the studies done previously, this skill was performed equally well by both control and interventional groups.

As with previous studies stated involving self-directed video and classroom teaching modules, our group-directed video instruction module was equally efficient in improving participants’ willingness to perform CPR after their training. However, the participants from the group-based video self-instruction who still felt reluctant to perform CPR cited the lack of proper training as the main cause for reluctance, perhaps due to lack of instructor guidance. This finding was somehow different compared to other studies where the video self-instruction participants felt the module was sufficient for them [34], [40]. Studies comparing the difference between methods of teaching science-based subjects in Malaysia and Germany concluded that our students have been used to the spoon-feeding approach in teacher-oriented learning during secondary school, resulting in a high school-university disconnect upon entering the university where they need to adapt to a self-directed style of learning, thus explaining the dependency upon the presence of a physical instructor [49], [50], [51], [52]. 

Participants who underwent traditional classroom instruction felt that the module equipped them with sufficient knowledge for them to initiate CPR, however, they cited the fear of contracting infectious diseases as the main reason for reluctance in performing CPR even after the training. Available data has shown that although the incidence of infection transmission to rescuers is low, the risks are not ignorable [48], [53], [54]. Some of the studies in which the participants did not put “fear of contracting infection” as the main factor of CPR reluctance included information regarding infection transmission and methods of minimizing risk during CPR [34]. Despite the heightened fear of contracting an airborne infection during this current pandemic, latest guidelines still encourages the performance of bystander CPR with proper safety precautions after carefully evaluating the benefits and risks, for both the victim and the rescuer [48]. In our modules, that theoretical information, however, was not included to save time. We recognized this as a limitation in our study thus for future courses, we suggest that such information is vital for inclusion. 

Another argument in our study is that we subjected the participants to view the video on a shared device as a group, and this may affect the outcome as some might not be able to catch up with the rest. However, we observed that all the interventional groups showed the capacity to work as a team to learn the module together. We chose this method of delivery to help cater to instances where the luxury of having a single device per person for video learning is not readily available. This might be helpful to prove that group-directed video learning for students or people in underprivileged or marginalized communities still may achieve optimal results [5], [25], [55], [56], [57].

## Limitation

Our sample population which is a small cohort comprising of first-year medical students in a single university is a limitation we acknowledge. Despite that, we have done our best to avoid familiarity bias and subject contamination bias among our participants. Being medical students, they might be more driven to learn about CPR compared to the members of the public. We however believe that it doesn’t affect their skill acquisition and performance. 

There is always a small possibility that prior knowledge of CPR was unevenly distributed among both groups, thus we have conveniently excluded students who have had any previous exposure to any form of CPR training so that their scores will not affect the results of this study. As our study emphasizes on skill performance rather than theoretical knowledge, we decided that a pre-test to gauge the ability of the participant to perform BLS would be redundant. BLS is a structured assessment that needs to be completed in a step-by-step fashion, thus without any prior knowledge or training, participants would definitely fail any form of pre-assessment when assessed using a structured rubric.

We didn’t include any evaluations to assess the theoretical knowledge pertaining to CPR as our study aims to compare skill competency acquisition between different delivery methods. This is because our modules were designed to emphasize less on the theoretical aspect of CPR to make it more appealing to the general public. However, we were unable to do the re-assessment of these skills gained over a period of time, due to unavoidable circumstances on our side. 

There is a concern that we allowed for non-competent participants to be evaluated for their willingness to perform CPR. However, we included them in the questionnaire to ascertain that willingness to perform CPR naturally comes after any training, regardless of the method of delivery. In real situations, however, we acknowledge the possible detrimental outcomes of such scenarios.

### Generalisability

Despite video learning methods not being a new method and has demonstrated success in self-directed studies, its utility in a group-directed setting is not yet properly explored. We believe that group members could provide valuable peer feedback during the video learning sessions thus leading to a better understanding of course material, compared to a single person using the same video content where this feedback is not available. However, this notion needs to be tested in future studies.

## Acknowledgement

The author would like to thank Universiti Sains Malaysia Health Campus and its Department of Emergency Medicine for providing the infrastructure and expertise for the study. A sincere vote of thanks to all the parties and individuals who have helped us during the study, notably during data collection and statistical analysis, and those who have had done similar studies before ours, is the cornerstone of this paper.

## Data and materials

Unpublished data or material from the study is available upon request from the authors via email.

## Funding

No sponsorship or financial support from any funding agency in the public, commercial, or non-profit sectors was awarded for the use of this study.

## Ethical statement

We obtained ethics approval from Human Research Ethical Committee, Universiti Sains Malaysia (Ref: USM/JEPeM/18060265). Written consent from the participants has been obtained for this study. 

### Human rights

The authors ensured there was no violation of human rights concerning to the study.

## Competing interests

The authors declare that they have no competing interests. 

## Supplementary Material

Additional material for this study

## Figures and Tables

**Table 1:**
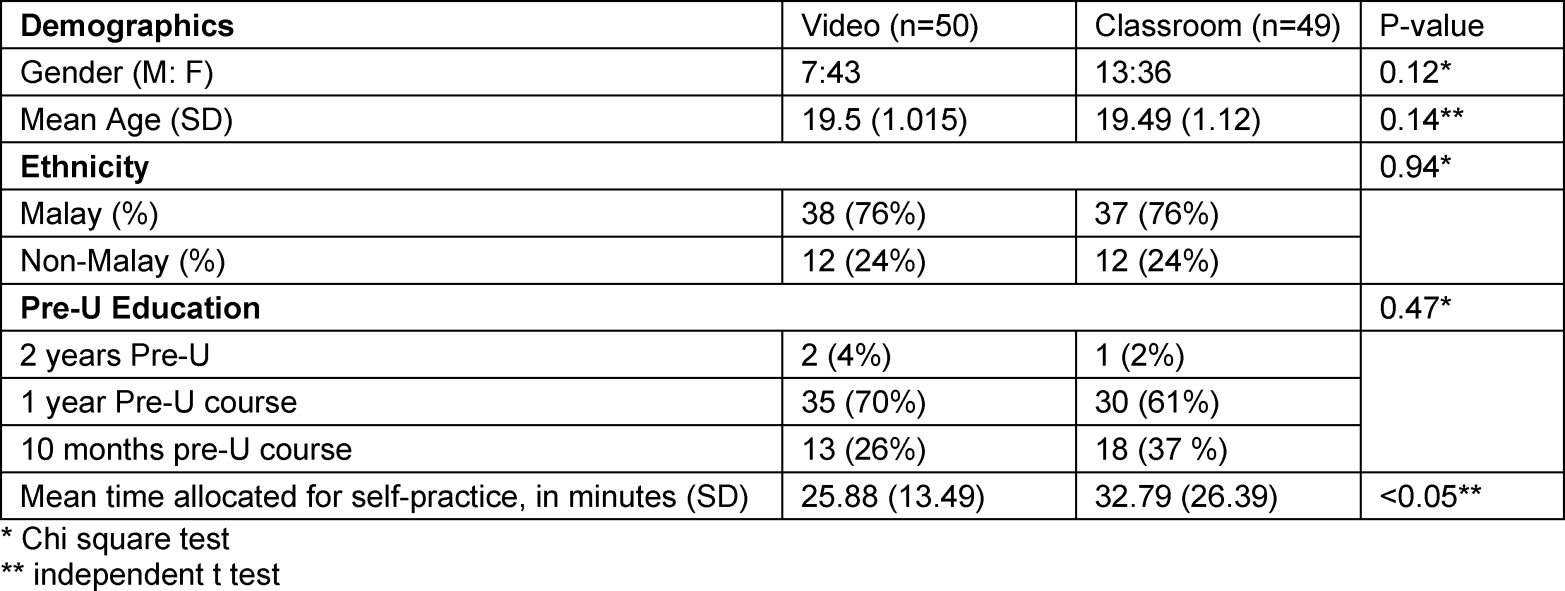
Participants’ demographic data according to modules

**Table 2:**
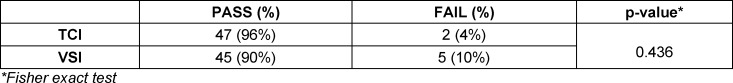
Pass-fail scores of participants in both modules

**Table 3:**
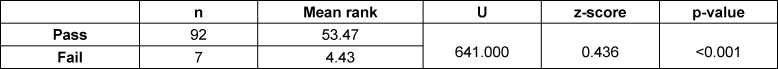
Mann-Whitney U Test comparing pass/fail outcomes and skill scores

**Table 4:**
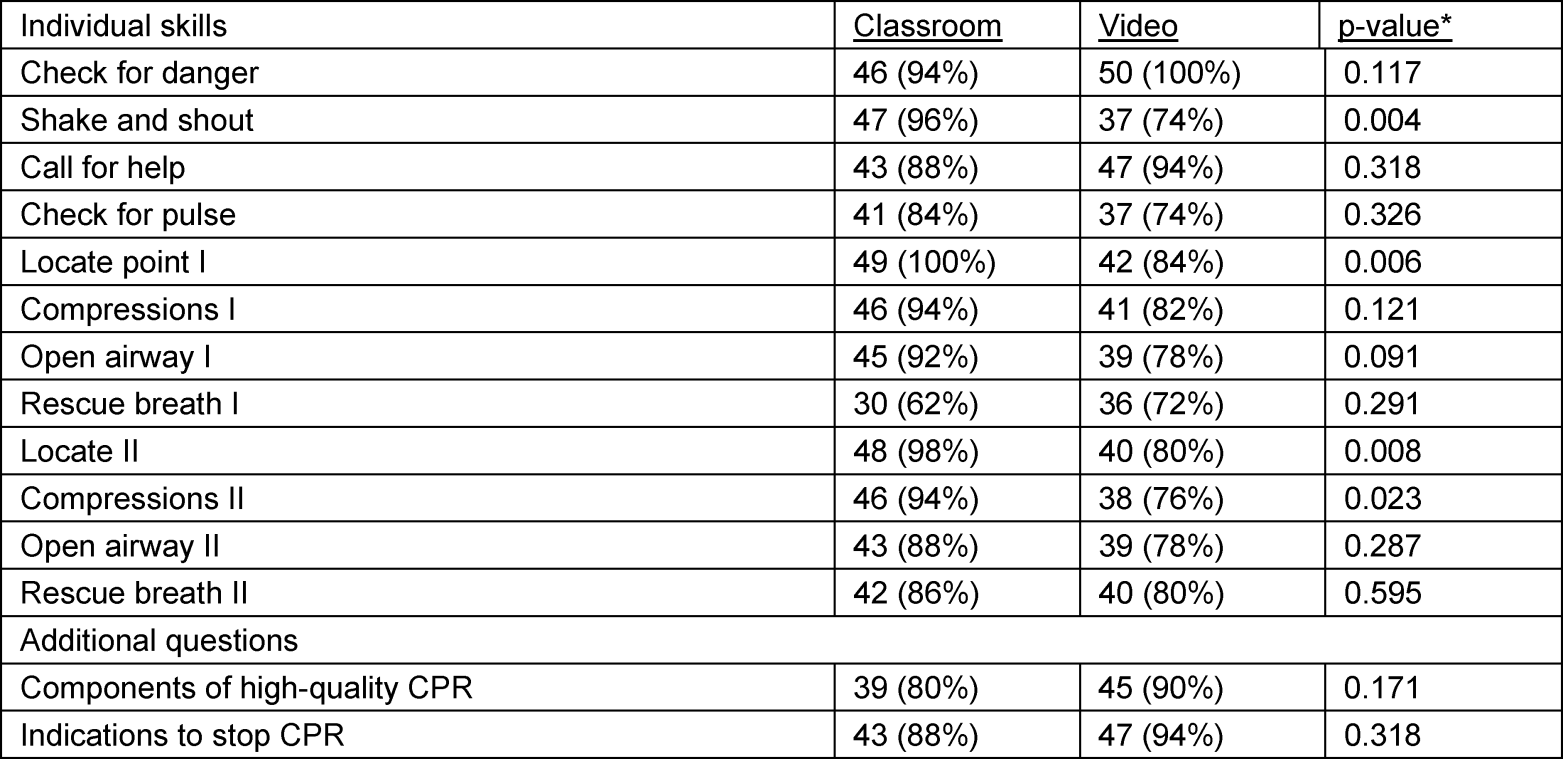
Association between module and individual CPR skills performance

**Table 5:**
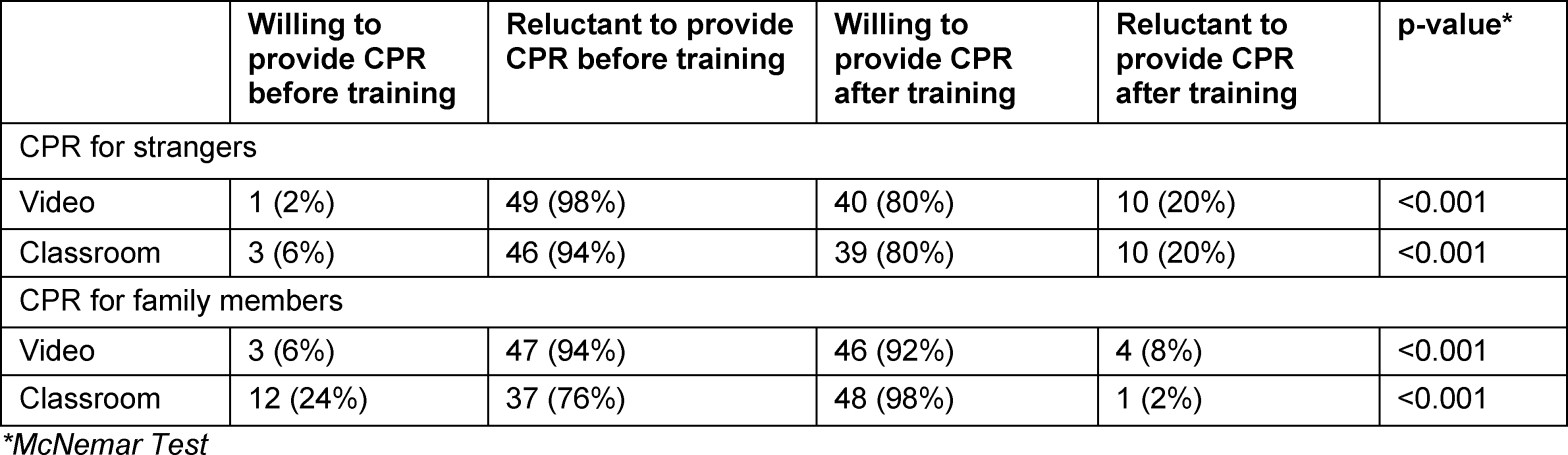
Association between willingness to provide CPR before training and after training
