# Supplementary material for: Comparing the effectiveness of a group-directed video instruction versus instructor-led traditional classroom instruction for learning cardiopulmonary resuscitation skills among first-year medical students: A prospective randomized controlled study
Source: GMS J Med Educ. 2022 Sep 15;39(4):Doc45. doi: 10.3205/zma001566 (PMC9585410; doi:10.3205/zma001566)
Supplement: Additional material for this study [file JME-39-45-s-001.pdf]

Attachment 1: Additional material for this study

Point A: Overall CPR score definition

Outstanding (5 points on the Likert score)
All skills were performed very well with no errors. CPR performed in this way is likely to be effective, and the victim would not be endangered

Very good (4 points on the Likert score)
All skills were performed competently, although improvement would be possible. Errors may be minor; most were corrected. No serious errors in technique or sequence were made. CPR performed in this way is likely to be effective, and the victim would not be endangered.

Competent (3 points on the Likert score)
Chest was compressed and ventilations resulted in chest rise. Skills were crude and sometimes failed to meet strict standards. Several steps may have been out of sequence. Some errors went uncorrected, although any serious errors were corrected. CPR performed in this way would probably be effective, and there would be no serious threat to the safety of the victim.

Questionably competent (2 points on the Likert score)
Some chest compressions were performed, and some ventilations resulted in chest rise. Skills were crude and often failed to meet the standard: serious errors were left uncorrected. There may have been serious errors in sequence or significant delays. CPR performed in this way might be effective; errors made might seriously affect the safety of the victim.

Not competent (1 point on the Likert score)
Efforts, if any, did not result in both chest rise and compression of chest. Skills were performed poorly or not at all; errors made might seriously endanger a victim. CPR performed in this way would probably not be effective, and the safety of the victim would be seriously threatened.

Point B: Individual skill checklist

1. Checks for danger
2. Checks unresponsiveness by touching manikin and speaking loudly
3. Calls for help or indicates help should be called
4. Checks carotid pulse for not more than 10 seconds
5. Locates compression position by feeling or baring chest and looking for the point.
6. Gives 30 compressions within 2 minutes
7. Opens airway using head-tilt/chin-lift
8. Attempts at least two breaths such that chest rises at least once and not more than twice
9. Locates compression position by feeling or baring chest and looking for the point.
10. Gives 30 compressions within 2 minutes
11. Opens airway using head-tilt/chin-lift
12. Attempts at least two breaths such that chest rises at least once and not more than twice

Point C: Skill definition

1. Checks for danger. Recognizes sources of danger within a given scenario.
2. Checks unresponsiveness by touching manikin and speaking loudly: Participant is close to the manikin, uses a loud voice, and touches the manikin.

   Sequence: the unresponsiveness check must precede any intervention including opening the airway.
3. Calls for help or indicates help should be called: Participant either simulates a phone call or indicates to the 'bystander' to call 911, call for an ambulance, or another clear instruction to make a phone call.

   Sequence: this must occur after a check of unresponsiveness and before starting chest compressions.
4. Checks carotid pulse for minimum of 5 seconds, maximum 10 seconds: Participant places fingers on Adam's Apple and slips fingers into the groove of uses an alternative method to establish correct position and then maintains position of fingers for at least 5 seconds. Count silently to yourself 1-1000, 2-1000, 3-1000, 4-1000, 5-1000. Short pulse checks do not count.

   Sequence: this must occur before any chest compressions
5. Locates compression position by feeling or baring the chest and looking: Participant finds position by using one of the following methods: (1) tracing outline of ribs and finding the place one finger above where the ribs come together (2) finding the xiphoid and placing two lines above it (3) baring the chest and visually finding a point on the sternum between the nipples.

6. Gives at 30 compressions per cycle at the correct location. Compressions must result in visible depression of the sternum and complete recoil of sternum. 18 seconds or less for 30 compressions.
7. Opens airway using head-tilt/chin-lift
8. Attempts at least two breaths such that chest rises at least once and not more than twice between every set of compression.
9. Locates compression position by feeling or baring the chest and looking, as in item (5)
10. Gives at 30 compressions per cycle at the correct location for second cycle. Compressions must result in visible depression of the sternum and complete recoil of sternum. Acceptable result is greater than 23 of 30 compressions.
11. Opens airway using head-tilt/chin-lift
12. Attempts at least 2 breaths such that chest rises at least once and not more than twice between every set of compression.

Point D: Overall rating score

OVERALL RATING SCORE<br>PARTICIPANT ID :	
RATING	CHOICE
5 EXCELLENT: All skills were performed very well with no errors. CPR performed in this way is likely to be effective, and the victim would not be endangered	
4 VERY GOOD: All skills were performed competently, although improvement would be possible. Errors may be minor; most were corrected. No serious errors in technique or sequence were made. CPR performed in this way is likely to be effective, and the victim would not be endangered.	
3 COMPETENT: Chest was compressed and ventilations resulted in chest rise. Skills were crude and sometimes failed to meet strict standards. Several steps may have been out of sequence. Some errors went uncorrected, although any serious errors were corrected. CPR performed in this way would probably be effective, and there would be no serious threat to the safety of the victim.	
2 QUESTIONABLY COMPETENT: Some chest compressions were performed, and some ventilations resulted in chest rise. Skills were crude and often failed to meet the standard: serious errors were left uncorrected. There may have been serious errors in sequence or significant delays. CPR performed in this way might be effective; errors made might seriously affect the safety of the victim.	
1 NOT COMPETENT: Efforts, if any, did not result in both chest rise and compression of chest. Skills were performed poorly or not at all; errors made might seriously endanger a victim. CPR may not have been performed. CPR performed in this way would probably not be effective, and the safety of the victim would be seriously threatened.	
EVALUATOR COMMENTS:	

Point E: Individual skills scoring sheet

INDIVIDUAL CPR SKILLS SCORING SHEET<br>PARTICIPANT ID :			
SKILL	ADEQUATE	INADEQUATE	COMMENTS
CHECK FOR DANGER			
SHAKE AND SHOUT			
CALL FOR HELP			
CHECKS PULSE FOR 10 SECONDS			
FIND CORRECT COMPRESSION POINT			
COMPRESSIONS (30)			
OPEN AIRWAY			
RESCUE BREATH (2) WITH CHEST RISE			
FIND CORRECT COMPRESSION POINT			
REPEATS COMPRESSIONS (30)			
REPEAT AIRWAY OPENING			
REPEATS BREATHING (2) WITH CHEST RISE			
ADDITIONAL QUESTIONS			
COMPONENTS OF HIGH QUALITY CPR			
INDICATIONS TO STOP CPR			

Point F: Evaluation questionnaire

**EVALUATION QUESTIONNAIRE**<br>*The information collected below will be used for research purposes only.*<br>*It will be accessed only by the investigators.*
**GENERAL INFORMATION** — Candidate No.:
Sex:<br>Age:<br>Race:<br>Previous learning institution:<br>Previous CPR course: YES/NO. If yes, last CPR course attended (year) : _______________
**ATTITUDE TOWARDS CPR : UNDERLINE YOUR CHOICE**
Were you comfortable in providing CPR to an **acquaintance, BEFORE** this study project? YES/NO<br>If no, state your reason:<br>Lack of training/Lack of confidence/Fear of infection<br>Others (please specify): ________________________
Were you comfortable in providing CPR to an **acquaintance, AFTER** this study project? YES/NO<br>If no, state your reason:<br>Lack of training/Lack of confidence/Fear of infection<br>Others (please specify): ________________________
Were you comfortable in providing CPR to a **stranger, BEFORE** this study project? YES/NO<br>If no, state your reason:<br>Lack of training/Lack of confidence/Fear of infection<br>Others (please specify): ________________________
Were you comfortable in providing CPR to a **stranger, AFTER** this study project? YES/NO<br>If no, state your reason:<br>Lack of training/Lack of confidence/Fear of infection<br>Others (please specify): ________________________
**Attitude towards learning material**
For Traditional Classroom Instruction (TCI) CPR course participants:<br>Comment upon the course: tick the box of your choice

**ASPECT**	**BAD**	**FAIR**	**GOOD**	**EXCELLENT**
Duration of course				
Content relevancy				
Time allocated for practice				

Total duration of self-practice during the course (minutes): ______________________________
Would you select TCI if you are allowed to choose again? Yes/No

Would you recommend TCI to others? Yes/No
Additional comments:
THANK YOU

For Video-Self Learning Instruction (VSI) CPR course participants:
Comment upon the course: tick the box of your choice

ASPECT	BAD	FAIR	GOOD	EXCELLENT
Duration of course				
Content relevancy				
Time allocated for self-practice				

Total duration of self-practice during the course (minutes): ____________________________
Would you select VSI if you are allowed to choose again? Yes/No
Would you recommend VSI to others? Yes/No
Additional comments:
THANK YOU

Point G: Evaluator script for use during assessment

All italicized portions are read or closely paraphrased to participants; roman text is instructions to evaluators. Text in brackets is read only to subjects evaluated during or at completion of a training class.

"We are in the process of evaluating and comparing several training methods. The best way to do this is to see what people learn [in our classes. The results of this evaluation will not affect whether or not you pass the course]"
"We have already cleaned the manikin [just like you did in class]. If you'd like, you can also clean it again yourself."
"This will be a realistic emergency situation; you should do whatever you think is necessary to save the victim's life. You will have to determine yourself what you need to do. For example, if you check the pulse on the manikin and there is no pulse, then you should do whatever you would do for a person who has no pulse. I won't be telling you about the condition of the victim once you start, and I won't be able to answer any questions. You can treat me like a bystander and tell me to do something that you would tell a bystander to do."
"If you make a mistake or forget to do something important you should not stop. Just do your best to correct the error. Please continue doing what you would do in an actual emergency until I tell you to stop."
"*Do you have any questions before we start?"*

If they ask any questions about CPR, you should not answer them. Tell them to :"*Do what you would do in an actual emergency.[You can ask your instructor that after we are done]."*

If they ask questions about what they are to do with the manikin tell them to: *"Check the manikin yourself and do what you think is needed to save a life."*

If they seem unsure, do your best to explain that they will be assessing the manikin and doing whatever is necessary. You can use examples, but do not tell them that they will be doing CPR.

"You have surveyed the scene to see if it is safe and to get some idea of what happened. Start now by doing what you would do in an actual emergency, and don't stop till I tell you to."

Attachment 1 to Mexmollen M, Ariff Arithra A, Junainah N, bin Tuan Hairulnizam TK, Tze Ping Pang N. *Comparing the effectiveness of a group-directed video instruction versus instructor-led traditional classroom instruction for learning cardiopulmonary resuscitation skills among first-year medical students: A prospective randomized controlled study.* GMS J Med Educ. 2022;39(4):Doc45. DOI: 10.3205/001566

Point H: Training program content guide

TCI CPR COURSE (approximately 4 hours)	VSI CPR COURSE (approximately 2 hours)
Lecture materials (approximately 2 hours)*<br>• Introduction to BLS<br>*lecture* (time varies)*<br><br>• Heart and lungs: anatomy and physiology *lecture* (time varies)*<br><br>• Causes of cardiac arrest<br>*lecture* (time varies)*<br><br>• Coronary heart disease<br>*lecture* (time varies)*<br><br>• Introduction to CPR<br>*lecture* (time varies)*<br><br>• Technical aspects of One-Rescuer CPR<br>- Check for danger<br>- Check for response<br>- Call for help<br>- Check for pulse<br>- Starts compressions<br>- Opens airway and gives rescue breaths<br>- Repeat for a complete cycle<br>- High-quality CPR<br>- When to stop CPR<br>- Recovery position<br>*lecture* (time varies)*<br><br>Mannikin practice<br>*demonstration*, *practice*<br>(approximately 2 hours)<br><br>Summary, Questions and Answers<br>*discussion* (time varies)	Video material (35 minutes in total)**<br><br>Initial assessment *(4’30)*<br>• Check for danger, *watch (1’)*<br>• Response, *watch (30”)*<br>• Call for help, *watch (40”)*<br>• Revision *practice (2’20”)*<br><br>Compression *(8’)*<br>• Check for pulse, *watch (15”)*<br>• Locate compression point, *watch (30”)*<br>• Proper posturing, *watch (30”)*<br>• 30 compressions, *watch (15”)*<br>• Revision, *practice (6’ 30”)*<br><br>Breaths *(6’30”)*<br>• Open airway, *watch (30”)*<br>• 2 rescue breaths, *watch (30”)*<br>• Revision, *practice (5’30”)*<br><br>Special considerations *(4’)*<br>• Stopping CPR, *watch (30”)*<br>• Recovery position *watch (1’30”)*<br>• Revision *practice (2’)*<br><br>Good quality CPR, *watch* (*2’)*<br><br>Revision of whole scenario in cycles, *practice (10’)*<br><br>***This video may be paused, replayed and repeated at any time as per needed by the group within a 2 hour time frame.*

Legend:
’ = minutes
” = seconds

Attachment 1 to Mexmollen M, Ariff Arithra A, Junainah N, bin Tuan Hairulnizam TK, Tze Ping Pang N. *Comparing the effectiveness of a group-directed video instruction versus instructor-led traditional classroom instruction for learning cardiopulmonary resuscitation skills among first-year medical students: A prospective randomized controlled study.* GMS J Med Educ. 2022;39(4):Doc45. DOI: 10.3205/001566

Point J: Consort Diagram: observational study

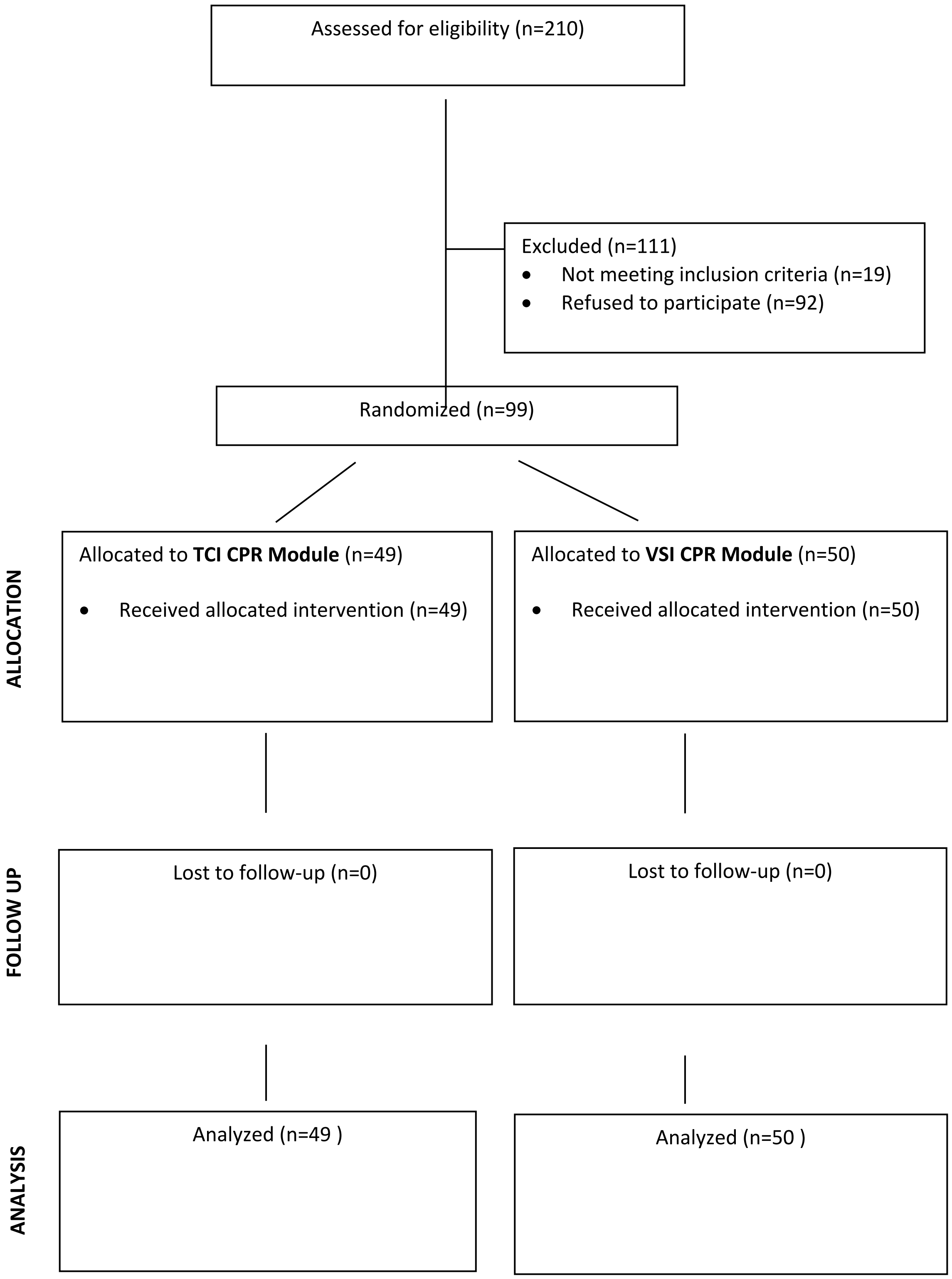

Attachment 1 to Mexmollen M, Ariff Arithra A, Junainah N, bin Tuan Hairulnizam TK, Tze Ping Pang N. *Comparing the effectiveness of a group-directed video instruction versus instructor-led traditional classroom instruction for learning cardiopulmonary resuscitation skills among first-year medical students: A prospective randomized controlled study.* GMS J Med Educ. 2022;39(4):Doc45. DOI: 10.3205/001566
